# A cluster of KPC-2 and VIM-2-producing *Klebsiella pneumoniae* ST833 isolates from the pediatric service of a Venezuelan Hospital

**DOI:** 10.1186/s12879-016-1927-y

**Published:** 2016-10-22

**Authors:** Aura Falco, Yusibeska Ramos, Esther Franco, Alegría Guzmán, Howard Takiff

**Affiliations:** 1 Laboratorio de Genética Molecular, Centro de Microbiología y Biología Celular, Instituto Venezolano de Investigaciones Científicas, Caracas, Venezuela; 2 Laboratorio B, Dirección de Energía y Ambiente, Instituto de Estudios Avanzados, Caracas, Venezuela; 3 Servicio de Laboratorio Clínico del anexo pediátrico “Dr. Rafael Tobías Guevara” del Complejo Hospitalario Universitario “Dr. Luis Razetti”, Barcelona, Venezuela

**Keywords:** *Klebsiella pneumoniae*, carbapenem, resistance, ST833

## Abstract

**Background:**

*Klebsiella pneumoniae* is a bacterial pathogen that has developed resistance to multiple antibiotics and is a major cause of nosocomial infections worldwide. Carbapenemase-producing *Klebsiella pneumoniae* have been isolated in many hospitals in Venezuela, but they have not been well-studied. The aim of this study was to characterize carbapenem-resistant *Klebsiella pneumoniae* isolates from the pediatric service of a hospital located in Anzoategui State, in the eastern part of Venezuela.

**Methods:**

Nineteen *Klebsiella pneumoniae* strains isolated in the hospital from April to July 2014 were evaluated phenotypically and molecularly for the presence of carbapenemases *bla*KPC, *bla*IMP and *bla*VIM. Molecular epidemiology was performed with Repetitive Extragenic Palindromic-PCR (REP-PCR) and Multilocus Sequence Typing (MLST). They were also studied for phenotypic and molecular resistance to a quaternary ammonium compound (QAC) disinfectant.

**Results:**

All 19 isolates contained both *bla*_VIM-2_ and *bla*_KPC-2_ genes, and the *bla*_KPC-2_ gene was associated with *Tn*4401b. All isolates were phenotypically sensitive to QACs and contained *qac*ΔE and *add*A2 genes typical of class 1 integrons. Analysis by REP-PCR and MLST showed that all isolates had identical profiles characteristic of sequence type ST833.

**Conclusion:**

All 19 strains are *bla*_VIM-2_ and *bla*_KPC_-_2−_producing ST833 *K. pneumoniae* sensitive to QACs. This analysis may help to understand the routes of dissemination and confirms that QAC disinfectants can be used to help control their spread.

**Electronic supplementary material:**

The online version of this article (doi:10.1186/s12879-016-1927-y) contains supplementary material, which is available to authorized users.

## Background

The emergence of plasmid-mediated carbapenem hydrolyzing β-lactamases and their spread amongst Gram-negative bacteria, especially *Klebsiella pneumoniae*, has become a serious threat for hospitalized patients worldwide. The two principal types of acquired carbapenemases are the molecular class B metallo-β-lactamases (MBLs) and the molecular class A *K. pneumoniae* carbapenemases (KPCs), both of which have been detected in many countries [1–4]. The *bla*_VIM_ gene encodes a transferable carbapenemase and is generally found within class 1 integrons. Although originally identified in *Pseudomonas aeruginosa*, VIM enzymes are now endemic within *Enterobacteriaceae* [5, 6]. The *bla*_KPC_ genes are usually located within *Tn*4401 [7], a *Tn*3-based transposon harbored on plasmids or in the chromosome. In recent years carbapenem-resistant *K. pneumoniae* has become a frequent cause of nosocomial infections in several hospitals in different Venezuelan cities [8], but the epidemiology, molecular epidemiology, clinical impact and carbapenemase genes of Venezuelan carbapenemase-producing *K. pneumoniae* have not been described. In this study we characterize 19 carbapenemase-producing *K. pneumoniae* isolates associated with nosocomial infections centered in the pediatric service of a large Venezuelan tertiary-care public hospital. All 19 isolates contained both *bla*_VIM-2_ and *bla*_KPC-2_ genes, were phenotypically sensitive to QACs and contained *qac*ΔE and *add*A2 genes typical of class 1 integrons. Analysis by REP-PCR and MLST showed that all isolates had identical profiles characteristic of sequence type ST833.

## Methods

### Hospital setting

The “Rafael Tobías Guevara” Pediatrics Department is part of the “Dr. Luis Razetti” Hospital, a large tertiary-care public hospital with 502 beds, located in Anzoátegui State, in the eastern part of Venezuela. The “Rafael Tobías Guevara” Pediatrics Department has 73 beds in the neonatal unit, 3 delivery rooms, 28 beds in the neonatal intermediate care unit and 12 beds in the neonatal intensive care unit. In 2014 nearly 26,000 women gave birth in this hospital, 30 % of whom were adolescents.

### Study population

The study was conducted from April to July 2014 and included all carbapenem-resistant *Klebsiella pneumoniae* strains isolated from pediatric patients hospitalized in the “Rafael Tobías Guevara” Pediatrics Department during this period. If there were more than one isolate from a patient, only the first was included in the study. Information on the source of the clinical specimen, the patient’s age, gender and hospital location was obtained from specimen records in the bacteriology laboratory and made irreversibly anonymous. The Bioethics Commission of the Instituto Venezolano de Investigaciones Científicas determined that the study did not require its approval.

### Bacterial strains and antibiotic susceptibility testing

The study included 19 *Klebsiella pneumoniae* strains isolated from April to July 2014 in the bacteriology laboratory of the “Rafael Tobías Guevara” Pediatrics Department. They all showed intermediate or full resistance to carbapenems according to the Clinical and Laboratory Standards Institute (CLSI) 2014 cutoff points [9]. The broader antimicrobial susceptibility profile of the *Klebsiella pneumoniae* strains was determined by the broth dilution method according to the CLSI guidelines for the following antibiotics: amikacin, tetracycline, chloramphenicol, amoxicillin/clavulanic acid, ampicillin, ampicillin/sulbactam, cefalotin, cefoxitin, ceftazidime, cefepime, piperacillin, piperacillin/tazobactam, aztreonam, cefoperazone/sulbactam, imipenem, meropenem, ciprofloxacin, and trimethoprim/sulfamethoxazole. Carbapenemase activity in all 19 isolates was confirmed by the Hodge test [9]. The disk diffusion assay with ertapenem and 3-aminophenyl boronic acid (400 *μ*g) was used to confirm the presence of KPC-type *β*-lactamases [10], and with ertapenem and EDTA to confirm the presence of metallo- *β*-lactamases in all 19 isolates.

### Detection of genes encoding β-lactamases and their genetic environment, integron cassettes and efflux pumps

Bacterial strains were grown on MacConkey agar and incubated overnight at 37 °C. One colony was resuspended in 100 μl of sterile distilled water and the bacteria were lysed by heating at 100 °C for 10 min. Cellular debris was removed by centrifugation at 13,000 g for 10 min and the supernatant was used as template DNA for PCR amplifications [11].

The carbapenemase genes *bla*_KPC_ [12] *bla*_IMP_ and *bla*_VIM_ [13] were amplified by PCR using the primers listed in Additional file 1: Table S1. The genetic environment of the *bla*_KPC_ genes was determined by PCR using previously described primers [14] specific for the *Tn*4401 transposon (Additional file 1: Table S1). To detect the *qac*ΔE gene encoding a putative efflux pump often found in class 1 integrons [15] as well as other integron cassettes [16], PCR was performed using primers specific for the 5′ and 3′ conserved integrons segments (Additional file 1: Table S1). PCR was also used for to detect putative efflux pumps *qac*A, *qac*B [15] and *qac*C genes [17] (Additional file 1: Table S1). The positive controls for the PCR reactions were characterized by phenotypic testing and PCR followed by sequencing of the relevant genes: a KPC-producing *K. pneumoniae* strain carrying a *bla*_KPC_ gene; a IMP-producing *P. aeruginosa* strain carrying a *bla*_IMP_ gene; a *P. aeruginosa* strain carrying a *bla*_VIM_ gene; and 4 isolates of *A. baumannii* resistant to QACs disinfectans carrying *qac*A, *qac*B, *qac*C and *qac*ΔE genes, respectively.

After PCR amplification, amplified fragments from the 19 clinical isolates were purified with Qiaquick PCR Spin columns (Qiagen) and sequenced in both forward and reverse directions (Macrogen, Korea) with the same primers used for PCR amplification. The sequences were compared with the National Center for Biotechnology Information (NCBI) (https://blast.ncbi.nlm.nih.gov/Blast.cgi) and Lahey databases (http://www.lahey.org/Studies/).

### Disinfectant susceptibility test in *Klebsiella pneumoniae* isolates

Susceptibility to Quaternary Ammonium Compounds (QACs) was determined using the quantitative suspension test for bactericidal activity, according to the protocol of Kawamura-Sato [18, 19]. The test was performed with the commercial disinfectant most commonly used in Venezuelan hospitals [20], which contains 10 % lauryl dimethyl benzyl ammonium bromide as the active QAC agent.

### Molecular genotyping

The genetic relationships amongst the 19 carbapenem-resistance isolates were determined by Repetitive Element Palindromic-PCR (REP-PCR) and Multi-Locus Sequence Typing (MLST). REP-PCR was performed using previously described primers REP1 and REP2 [21] (Additional file 1: Table S1). MLST was performed using the methodology described by Diancourt et al. [22] (Additional file 1: Table S1). After PCR amplification, the fragments were sequenced with the amplification primers in the forward and reverse directions by Macrogen, Korea. Allele numbers and sequence types (STs) were assigned by the *Klebsiella pneumoniae* MLST web site (http://bigsdb.pasteur.fr/klebsiella/klebsiella.html).

### Plasmid analysis

Plasmid DNA was extracted by the Kieser extraction method [23] and analyzed by gel electrophoresis in 0.7 % agarose.

## Results

### Clinical and epidemiological characteristics

We analyzed 19 carbapenem-resistant *K. pneumoniae* strains isolated from clinical specimens of an equal number of patients in the “Rafael Tobías Guevara” Pediatrics Department located in the “Dr. Luis Razetti” Teaching Hospital. The patients’ demographic and clinical characteristics are summarized in Table 1. All infections were health care associated. Fourteen patients (*n* = 14/19, 73.7 %) were newborns and 12 (*n* = 12/19, 63.1 %) were male. The most common infection sites were bloodstream (*n* = 15/19, 79 %) and bronchial secretions (*n* = 2/19, 10.5 %) (Table 1).

**Table 1:** Clinical characteristics of patients infected by carbapenem-resistant *K. pneumoniae* isolates from a Venezuelan Hospital

	No. (%) of isolates
Characteristic	Total no.
Gender	
Female	7 (36.9 %)
Male	12 (63.1 %)
Age	
Newborn (0–6 days)	14 (73.7 %)
Lower infant (1–12 months)	2 (10.5 %)
Higher infant (1–2 years)	2 (10.5 %)
School (5–10 years)	1 (5.3 %)
Infection site	
Bloodstream	15 (79 %)
Bronchial secretion	2 (10.4 %)
Catheter	1 (5.3 %)
Lesion secretion	1 (5.3 %)
Hospitalization área	
Neonatal	12 (63.2 %)
Internal medicine	2 (10.5 %)
Intensive care	2 (10.5 %)
Surgery	3 (15.8 %)

### Antibiotic susceptibility testing of *K. pneumoniae* isolates

All 19 isolates were resistant to trimethoprim-sulfamethoxazole, imipenem and meropenem, but testing for resistance to other antibiotics revealed seven resistance profiles. Profiles 2 and 5 were the most common with four isolates each, followed by profiles 1 and 4 with three isolates each (Table 2). The isolates in profile 7, the most resistant, were not sensitive to any of antibiotics tested, while profile 2 isolates were the most sensitive, showing resistance only to ciprofloxacin (Table 2). The percentages of isolates resistant to other antibiotics were: amikacin, 63.2 %; ciprofloxacin, 94.7 %; tetracycline, 84.2 %; chloramphenicol, 42.1 %; and tigecycline 15.8 %.

**Table 2:** Antibiotic susceptibility profiles of carbapenem-resistant *K. pneumoniae* isolates from a Venezuelan Hospital

Profile:Isolates	CM	AK	CIP	TE	TYG	% of isolates belonging profile
1: ANZ2, ANZ4, ANZ6	S	S	R	S	S	15.8
2: ANZ8, ANZ11, ANZ12, ANZ13	S	S	R	R	S	21.0
3: ANZ10	S	R	S	R	S	5.3
4: ANZ1, ANZ3, ANZ19	S	R	R	R	S	15.8
5: ANZ5, ANZ7, ANZ9, ANZ14	R	R	R	R	S	21.0
6: ANZ16	R	R	R	R	-	5.3
7: ANZ17, ANZ18, ANZ21	R	R	R	R	R	15.8

All strains were resistant to imipenem, meropenem, and trimethoprim-sulfamethoxazole

*CM* chloramphenicol, *AK* amikacin, *CIP* Ciprofloxacin, *TE* tetracycline, *TGC* tigecycline, (−) no data

### Detection of genes encoding β–lactamases

Using PCR, both the *bla*_KPC-2_ and *bla*_VIM-2_ genes were detected in 100 % (*n* = 19/19) of the isolates, while the *bla*IMP gene could not be amplified from any isolate.

### Genetic environment of *bla*_KPC_ gene

**Fig. 1:**
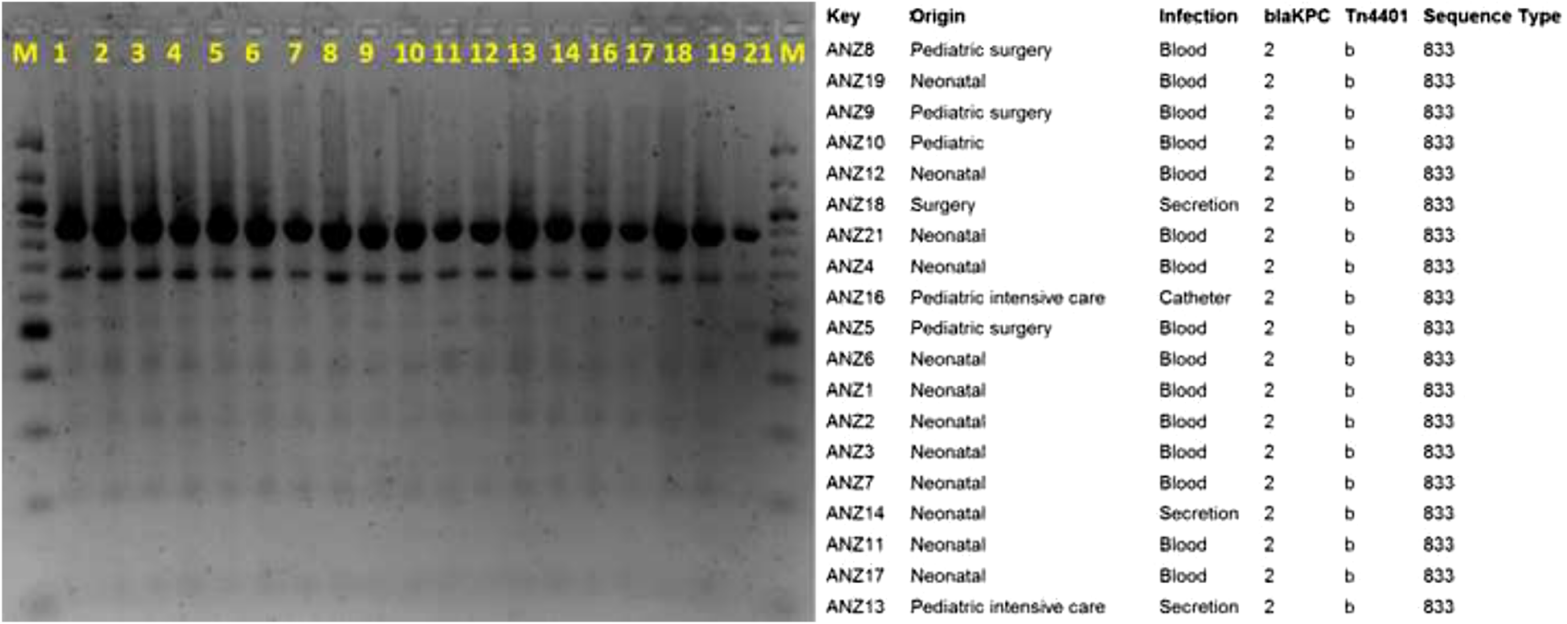
REP-PCR profiles of 19 isolates of *bla*_VIM-2_ and *bla*_KPC-2_-coharboring *K. pneumoniae* from a Public Venezuelan Hospital. Bands were visualized with ethidium bromide staining on a 1.0 % TBE agarose gel. M: Molecular Weight Marker 100 bp ladder (New England Biolabs®), line 1: ANZ1, line 2: ANZ2, line 3: ANZ3, line 4: ANZ4, line 5: ANZ5, line 6: ANZ6, line 7: ANZ7, line 8: ANZ8, line 9: ANZ9, line 10: ANZ10, line 11: ANZ11, line 12: ANZ12, line 13: ANZ13, line 14: ANZ14, line 16: ANZ16, line 17: ANZ17, line 18: ANZ18, line 19: ANZ19, line 21: ANZ21. The table to the right illustrates results from the *bla*_KPC_ sequence analysis, MLST and sequence analysis of the nonconserved region of the *Tn*4401 element

The *bla*_KPC_ gene is generally associated with a *Tn*3-based transposon, *Tn*4401, composed of a transposase gene, a resolvase gene, the *bla*_KPC_ gene and two insertion sequences (*IS*Kpn6 and *IS*Kpn7) [7]. Six isoforms of *Tn*4401 have been identified which differ by various deletions upstream of the *bla*_KPC_ gene [24, 25]. Amplification of the regions flanking the *bla*_KPC-2_ genes with *Tn*4401 specific primers [14] produced a 703-bp PCR fragment from all 19 isolates, consistent with the *Tn*4401 variant “b” [14] (Fig. 1). The *IS*Kpn6 and *tnp*A genes were also amplified separately from all 19 isolates, but the inverted repeat sequences of *Tn*4401 could not be amplified with primers specific for this region [7, 14, 26] (Additional file 1: Table S1).

### Detection of genes located in class 1 integrons

Amplification and sequencing with primers specific for class 1 integrons detected *qac*ΔE and *add*A2 genes in all 19 isolates. The *add*A2 gene encodes an aminoglycoside adenyltransferase that confers streptomycin resistance, while *qac*ΔE gene encodes a truncated Quaternary Ammonium Compounds resistance protein.

### Disinfectant susceptibility and molecular detection of the *qac*A, *qac*B, and *qac*C genes

All 19 isolates were susceptible to a disinfectant containing QAC agent lauryl dimethyl benzyl ammonium bromide. The *qac*A, *qac*B and *qac*C genes, implicated in resistance to this type of disinfectants, could not be amplified from any of the isolates.

### Molecular genotyping

The REP-PCR technique produced very similar patterns from all 19 *K. pneumoniae* isolates. PCR amplification and sequencing of the seven genes [22] used for MLST produced identical profiles from all 19 strains. Comparison with the *Klebsiella pneumoniae* MLST website (http://bigsdb.pasteur.fr/klebsiella/klebsiella.html) revealed that this MLST profile is characteristic of sequence type, ST833 (100 %, *n* = 19/19) (Fig. 1).

### Plasmid analysis

**Fig. 2:**
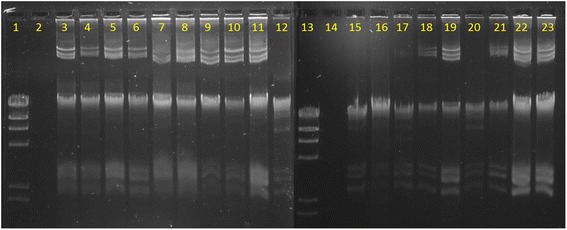
Plasmidic DNA profile of 19 isolates from a Public Venezuelan Hospital. Bands were visualized with ethidium bromide staining on a 0.7 % TBE agarose gel. Line 1: 100 bp ladder (New England Biolabs®), line 2: *Escherichia coli* XL1-Blue (negative control), line 3: ANZ1, line 4: ANZ2, line 5: ANZ3, line 6: ANZ4, line 7: ANZ5, line 8: ANZ6, line 9: ANZ7, line 10: ANZ8, line 11: ANZ9, line 12: ANZ10, line 13: 100 bp ladder (New England Biolabs®), line 14: *Escherichia coli* XL1-Blue (negative control), line 15: ANZ11, line 16: ANZ12, line 17: ANZ13, line 18: ANZ14, line 19: ANZ16, line 20: ANZ17, line 21: ANZ18, line 22: ANZ19, line 23: ANZ21

Plasmid DNA was isolated from all 19 isolates, and the plasmid profiles are shown in Fig. 2. It appears that there are some similarly sized plasmids that are present in almost all of the 19 isolates. These shared plasmids could carry the located *bla*_KPC-2_, *bla*_VIM-2_, *add*A2 and *qacΔ*E genes, which have been commonly found in class 1 integrons within transposons carried on plasmids [27–30].

## Discussion

We report 19 carbapenem reistant *K. pneumoniae* strains isolated from the pediatrics service of a hospital in Venezuela that co-harbor both *bla*_KPC_ and *bla*_VIM_ genes. All 19 strains appear to have identical Rep-PCR profiles, and by MLST all appear to belong to ST833, suggesting that this resistant strain has become endemic in the pediatric service, especially in the neonatal units of this hospital, where 12 of the strains were isolated. The limited patient information available and irreversibly anonymous nature of the strains did not permit retrospective analysis of the patients to exclude the possibility that some represented colonization or transient carrier states with KPC-producing *K. pneumoniae*. This seems unlikely, as 15 of the 19 strains were isolated from blood cultures, suggesting they were associated with severe infections. Cross contamination also seems unlikely as the isolates showed seven different profiles of resistance to other antibiotics.

There are no official data available about epidemiological monitoring of bacteria causing nosocomial infections in Venezuelan hospitals. The only surveillance program that provides data about antimicrobial resistance in Venezuela is PROVENRA (http://provenra.cloudapp.net/), a private initiative with information on antibiotic resistance from 1998 to 2012. The information is collected from microbiology laboratories in 46 hospitals nationwide, but does not include the “Dr. Luis Razetti” hospital studied in the present report. According to data reported for 2012 on the PROVENRA webpage, 72 % of *K. pneumoniae* isolated in neonatology services were resistant to ertapenem and 65 % were resistant to meropenem and imipenem (http://provenra.cloudapp.net/). This high prevalence of resistance was confirmed in our study.

Other studies have described carbapenem resistance in Venezuela. Fernández-Canigia and Dowziki in 2012 [31] presented data from the Latin American region on Gram-negative isolates used in the Tigecycline Evaluation and Surveillance Trial (T.E.S.T.). They describe decreasing susceptibility to carbapenems among ESBL producing *K.* pneumoniae in the Latin America countries studied, but found that 90.3 % of Venezuelan strains were sensitive to meropenem. Jones et al. [32] reported the results of a resistance surveillance program monitoring antimicrobial susceptibility patterns in Latin America in which only 15 % of *Klebsiella* isolates were carbapenem-resistant. Finally, Kazmierczak et al. [33] analyzed Gram-negative pathogens collected from 40 countries, including Venezuela, as part of a global surveillance study in 2012–2014. Carbapenem non-susceptible *Enterobacteriaceae* were characterized for *bla* genes encoding MBLs and serine β-lactamases variants with PCR and sequencing. In the strains from Venezuela there was one isolate of *K. pneumoniae* containing NDM-1 and one isolate of *P. aeruginosa* carrying VIM-2 [33].

The KPC-2 allele, one of 21 variants of the *bla*_KPC_ gene, was found in all our 19 *K. pneumoniae* isolates and is one of the most extensively distributed worldwide [34], including in the South American countries of Colombia [35, 36] Brazil [37–40] Argentina [41, 42]. In Venezuela [43, 44], a KPC-2-producing *K. oxytoca* was isolated from a pediatric patient in the state of Mérida [44], but to our knowledge, KPC genes have not been previously reported in Venezuelan *K. pneumoniae* isolates.

In addition to the KPC-2 gene, all of our 19 isolates also carried the VIM-2-carbapenemase. The *bla*_VIM_ gene is extensively distributed worldwide, with VIM-2 the most widespread variant [45]. Endemicity of VIM enzymes has been reported in Greece, Taiwan, and Japan [46, 47], although outbreaks and single strains of VIM producers have been reported in many other countries including the Latin American countries of México, Argentina, Colombia and Venezuela [45]. In Venezuela the *bla*_VIM_ gene was previously found in clinical isolates of *Pseudomonas aeruginosa* [48, 49], and Marcano et al. [50] reported VIM-producing carbapenem-resistant *K. pneumoniae* isolated from the urine of a 7-year-old girl hospitalized at the “Hospital de Niños J. M. de los Ríos” in Caracas, Venezuela.

Strains carrying both *bla*_KPC_ and *bla*_VIM_ carbapenemases have been previously reported in Greece [1, 51–54] Colombia [55], Germany [56], Italy [6], and Spain [57]. In Venezuela the two carbapenemases have only been reported together in a multiply resistant strain of *Enterobacter cloacae* [58] isolated from the urine of a 83-year-old patient in a hospital in the city of Cumaná, which is only about 90 miles from hospital we studied. It might be interesting to know whether both the *E. cloacae* and *K. pneumoniae* isolates could be carrying these carbapenemase genes within the same transferable genetic context.

Class 1 integrons are the most common integron type present in clinical isolates of the *Enterobacteriaceae*, and are increasingly detected in isolates of *K. pneumoniae*. The gene cassettes most frequently identified within class 1 integrons in *Enterobacteriaceae* are those encoding resistance to streptomycin (*aadA*) and trimethoprim (*dfrA*) [59, 60]. By PCR we found that 19 *K. pneumoniae* strains contained the *add*A2 gene within the 5′CS-3′CS region of the integron. We did not look for the presence of the *dfrA* gene, but all 19 strains were resistant to trimethoprim-sulfamethoxazole. The *qacΔ*E gene, which has also been associated with class 1 integrons [61] was amplified from all 19 isolates.

KPC genes are often found within a transposon-associated element, *Tn*4401 [7]. *Tn*4401 possesses genes encoding a transposase (*tnp*A) and a resolvase (*tnp*R), and has been characterized as an active transposon that is able to mobilize the *bla*_KPC_ genes at high frequency without target specificity [7, 25]. In all 19 strains in this study the *bla*_KPC-2_ genes appeared to be within a *Tn*3-based structure consistent with the *Tn*4401 isoform ‘b’ [62], which has been observed in Greece [63], Colombia [14], Brazil [64] and the USA. Similar to the report by Pereira et al. [64] the inverted repeat sequences of the flanking regions were not amplified in our isolates, suggesting that their insertion sites may be different from those of *K. pneumoniae* YC described by Naas et al. [7].

The KPC and VIM genes are generally found on plasmids [65], and the plasmids profiles from all 19 strains contained similar bands, suggesting that the integron containing the carbapenemase genes could be present within a transposable element on a common plasmid, but further studies are needed to confirm this possibility.

All 19 isolates evaluated in this study demonstrated similar patterns with REP-PCR analysis, and by MLST all belonged to ST833, a genotype that has only been reported in Israel [28] and Trieste, Italy [29]. Interestingly, the ST833 *K. pneumoniae* strain isolated in the Trieste Pediatric Hospital was from a blood culture of a 3-year-old patient transferred from a Venezuelan hospital to undergo marrow transplantation [29].

We have not found any previous studies describing MLST characterization of *K. pneumoniae* strains isolated in Venezuela, but we have used MLST to analyze the strains of KPC-producing *K. pneumoniae* in a few other hospitals in the country (manuscripts submitted). Although the other hospitals always contained a variety of sequence types, we found isolates belonging to ST833 in hospitals in two other Venezuelan states--Zulia and the Capital District. These are in the west and center of the country, respectively, and distant from hospital studied in the current report, which is located in the eastern state of Anzoátegui. From our albeit limited sampling, it appears that the carbapenem resistant *K. pneumoniae* ST833 strain could be extensively distributed throughout the country. Perhaps a Venezuelan patient acquired the ST833 strain as an infection or colonization while hospitalized in Israel and then returned to Venezuela, where it subsequently disseminated throughout the country with the movements of patients and health care staff.

ST833 is part of the 258 clonal complex, and the ST833 allelic profile (3-3-1-1-1-1-12) differs from ST258 only in the *ton*B allele. The clonal complex CC258 is the dominant and most successful KPC producing strains as has spread widely and rapidly across the world [30, 66], although the reasons for its apparent advantage have yet to be completely explained facilitated by its production of proteins involved in cell motility, secretion and DNA repair and modification [67–69].

Disinfectants containing QACs are commonly used in Venezuelan hospitals, and therefore the presence of QAC resistance would complicate efforts to reduce the prevalence of this ST833 strain. Fortunately, all strains were phenotypically sensitive to the QAC disinfectant and we could not amplify the *qac*A, *qac*B [15] or *qac*C genes [16, 17] which have all been associated with QAC resistance. Nevertheless, the use of QAC disinfectants is clearly only a minor part of the measures that are required to control nosocomial infections.

Venezuelan medical personnel are keenly aware that carbapenem-resistant *K. pneumoniae* have become a frequent cause of nosocomial infections in several hospitals in different Venezuelan cities [8]. We hope that the molecular epidemiology provided by this study can aid in tracking the presence and dissemination of the strains involved, and thereby contribute to the vigilance and surveillance required to effectively treat them and to reduce their presence.

## Conclusions

Molecular characterization of 19 carbapenem-resistant *K. pneumoniae* strains isolated from pediatric patients in a large public hospital in Anzoátegui, Venezuela, revealed that all contained the *bla*_KPC-2_ and *bla*_VIM-2_ genes, encoding serine and metallo carbapenemases, respectively. MLST analysis showed that all 19 strains belong to ST833, suggesting an alarming endemic presence of this strain throughout the hospital’s pediatric service, but particularly in the neonatal units.

## Additional file

Additional file 1: Table S1. Oligonucleotides used for PCR of carbapenemase-resistant *Klebsiella pneumoniae* isolates from a Hospital in Venezuela. (DOC 77 kb)

## Abbreviations

AK: Amikacin
bp: Base-pair
CC: Clonal complex
CIP: Ciprofloxacin
CLSI: Clinical and Laboratory Standards Institute
CM: Chloramphenicol
CS: Conserved-segment
DNA: Deoxyribonucleic acid
Dr: Doctor
IMP: Imienemase
IS: Insertion sequences
KPC: *Klebsiella pneumoniae* Carbapenemase
MBL: Metallo-β-lactamase
MLST: Multi-Locus Sequence Typing
NCBI: National Center for Biotechnology Information
NDM: New Delhi Metallo-β-lactamase
PCR: Polymerase chain reaction
QAC: Quaternary Ammonium Compounds
REP-PCR: Repetitive Element Palindromic - Polymerase Chain Reaction
ST: Sequence type
TE: Tetracycline
TGC: Tigecycline
Tn: Transposon
UPMG: Unweighted pair group method
VIM: Verona Integron-ecoded Metallo-β-lactamase
