# Supplementary material for: A cluster of KPC-2 and VIM-2-producing Klebsiella pneumoniae ST833 isolates from the pediatric service of a Venezuelan Hospital
Source: BMC Infect Dis. 2016 Oct 22;16:595. doi: 10.1186/s12879-016-1927-y (PMC5075218; doi:10.1186/s12879-016-1927-y)
Supplement: Additional file 1: Table S1. — Oligonucleotides used for PCR of carbapenemase-resistant Klebsiella pneumoniae isolates from a Hospital in Venezuela. (DOC 77 kb) [file 12879_2016_1927_MOESM1_ESM.doc]

**S1 Table.** Oligonucleotides used for PCR of carbapenemase-resistant *Klebsiella pneumoniae* isolates from a Hospital in Venezuela.

| **Primer name** | **Primer sequences** | | **Product size (bp)** | **Annealing temp. (°C)** | **References** |
| --- | --- | --- | --- | --- | --- |
| **Carbapenemases** | | | | | |
| KPC-F  KPC-R | | 5-ATGTCACTGTATCGCCGTCT-3  5-TTTTCAGAGCCTTACTGCCC-3 | 894 | 53 | Bradford, 2004 |
| IMP-F  IMP-R | | 5-GGAATAGAGTGGCTTAAYTCTC-3  5-GGTTTAAYAAAACAACCACC-3 | 232 | 52 | Poirel, 2011 |
| VIM-F  VIM-R | | 5-GATGGTGTTTGGTCGCATA-3  5-CGAATGCGCAGCACCAG-3 | 390 | 52 | Poirel, 2011 |
| **QAC genes** | |  |  |  |  |
| *qac*A-F  *qac*A-R | | 5-GCTGCATTTATGACAATGTTTG-3  5-AATCCCACCTACTAAAGCAG-3 | 629 | 45 | Wang, 2008 |
| *qac*B-F  *qac*B-R | | 5-CTATGGCAATAGGAGATATGGTGT  5-CCACTACAGATTCTTCAGCTACATG | 416 | 45 | Wang, 2008 |
| *qac*C-F  *qac*C-R | | 5-AAACAATGCAACACCTACCACT-3  5-AACGAAACTACGCCGACTATG-3 | 157 | 51 | Mayer, 2001 |
| **Class 1 integron** | |  |  |  |  |
| 5´CS  3´CS | | 5-GGCATCCAAGCAGCAAG-3  5-AAGCAGACTTGACCTGA-3 | Variable | 60 | Levesqué, 1995 |
| *qacΔ*E-F  *qacΔ*E-R | | 5-TAGCGAGGGCTTTACTAAGC-3  5-ATTCAGAATGCCGAACACCG-3 | 300 |  | Wang, 2008 |
| ***Tn*4401** | | | | | |
| 4281  4714 | 5-GGCACGGCAAATGACTA-3 | | 651 | 55 | Cuzon, 2011 |
|  | 5-GAAGATGCCAAGGTCAATGC-3 | |  |  |  |
| EcoRIout  3´YCEnd | 5-CACCCGACCTGGACGAACTA-3 | | 252 | 55 | Cuzon, 2011 |
|  | 5-GCATCAAACGGAAGCAAAAG-3 | |  |  |  |
| 3781L  3098U | 5-CACAGCGGCAGCAAGAAAGC-3 | | Variable | 55 | Cuzon, 2011 |
|  | 5-TGACCCTGAGCGGCGAAAGC-3 | |  |  |  |
| 905L  816U | 5-GCGACCGGTCAGTTCCTTCT-3 | | 199 | 55 | Cuzon, 2011 |
|  | 5-CACCTACACCACGACGAACC-3 | |  |  |  |
| 141R-6  5´endYC | 5-TCACCGGCCCTCACCTTTGG-3 | | 463 | 55 | Cuzon, 2011 |
|  | 5-CTTAGCAAATGTGGTGAACG-3 | |  |  |  |
| **REP-PCR** | | | | | |
| REP1 | 5-IIIGCGCCGICATCAGGC-3 | | Variable | 50 | Versalovic, 1991 |
| REP2 | 5-ACGTCTTATCAGGCCTAC-3 | |  |  |  |
| **MLST** | | | | | |
| *rpo*B F Vic3  *rpo*B R Vic2 | 5-GGCGAAATGGCWGAGAACCA-3 | | 1070 | 50 | Diancourt, 2005 |
|  | 5-GAGTCTTCGAAGTTGTAACC-3 | |  |  |  |
| *mdh* F 130  *mdh* R 867 | 5-CCCAACTCGCTTCAGGTTCAG-3 | | 757 | 50 | Diancourt, 2005 |
|  | 5-CCGTTTTTCCCCAGCAGCAG-3 | |  |  |  |
| *pgi* F 1R  *pgi* R 1F | 5-GAGAAAAACCTGCCTGTACTGCTGGC-3 | | 718 | 50 | Diancourt, 2005 |
|  | 5-CGCGCCACGCTTTATAGCGGTTAAT-3 | |  |  |  |
| *gap*A F 173  *gap*A R 181 | 5-TGAAATATGACTCCACTCACGG-3 | | 663 | 60 | Diancourt, 2005 |
|  | 5-CTTCAGAAGCGGCTTTGATGGCTT-3 | |  |  |  |
| *pho*E F 604.1  *pho*E R 604.2 | 5-ACCTACCGCAACACCGACTTCTTCGG-3 | | 603 | 50 | Diancourt, 2005 |
|  | 5-TGATCAGAACTGGTAGGTGAT-3 | |  |  |  |
| *ton*B 1F  *ton*B 2R | 5-CTTTATACCTCGGTACATCAGGTT-3 | | 540 | 45 | Diancourt, 2005 |
|  | 5-ATTCGCCGGCTGRGCRGAGAG-3 | |  |  |  |
| *inf*B 1F  *inf*B 1R | 5-CTCGCTGCTGGACTATATTCG-3 | | 463 | 50 | Diancourt, 2005 |
